# A great race of drinkers? Irish interpretations of alcoholism and drinking stereotypes, 1945–1975

**DOI:** 10.1017/mdh.2020.51

**Published:** 2021-01

**Authors:** Alice Mauger

**Affiliations:** School of History, University College Dublin, Belfield, Dublin, Ireland

**Keywords:** Alcoholism, Alcohol, Problem drinking, Stereotype, Ireland, Irish culture

## Abstract

From the 1930s, psychiatrists and sociologists documented the prevalence of Irish alcohol-related psychiatric admissions in the United States. These studies seemed to suggest that the Irish, as a race, had a remarkable relationship with drink, therefore reinforcing the enduring ‘drunken Irish’ stereotype. By the 1960s, the alleged Irish susceptibility to alcoholism gained increasing attention from researchers and officials in Ireland itself. Significantly, this renewed awareness coincided with a shift in Ireland’s place on the international landscape and was intertwined with the broader social, cultural and political environment. While anxieties about the apparently rising incidence of alcoholism and alcohol-related harm were not unique to Ireland, the specific cultural meanings attached to excessive drinking in a nation internationally renowned for this problem mapped onto shifting international frameworks, informing medical perceptions and shaping policy developments. This article explores expert and official interpretations of alcoholism and the ‘drunken Irish’ stereotype from 1945 to 1975. This period saw a number of important developments, including the introduction of the Irish Mental Treatment Act of 1945, the establishment of the Irish National Council on Alcoholism in 1966 and the creation of specialist alcohol treatment facilities in several psychiatric hospitals. In the same era, the contexts for understanding problem drinking began to shift from the disease concept of alcoholism towards the public health perspective on alcohol. As will be argued, in Ireland, these frameworks were coloured by concerns that social and cultural factors were contributing to rising levels of alcohol consumption and psychiatric admissions for alcoholism.

## Article

In 1962, the *Journal for the Irish Medical Association* featured an article on cultural influences in psychiatric illness in the Irish. It was penned by Dr Dermot Walsh, an Irish psychiatrist working at St James’ Hospital in Leeds, and included a lengthy treatise on what he termed the Irish ‘cultural environment’. Among a range of factors of perceived importance, Walsh listed the persistence of ancient folk customs; an ‘immense capacity for superstition and magical belief’; the consequences of warfare and material hardship since 1550; the cataclysmic impact of the Great Famine of the 1840s; and the combined effects of the Irish mother’s dominance, Irish father’s diminished authority and Irish son’s deeply ambivalent and conflict-laden maternal relationship resulting, he reasoned, in ‘some modification of the classical Oedipal situation’. Predictably, he also presented a detailed discussion of the alleged Irish predisposition to alcoholism, both at home and abroad. Walsh premised that the Irish were:

in a general sense freer in their use of alcohol than most nationalities. There seems little doubt that drinking plays a large part in dealing with many deeper frustrations and conflicts … Alcohol, too, has in high degree the effect of inducing and enhancing the fantasy life which is so inherently a part of Irish cultural heritage.^1^

Walsh went on to become a highly influential figure in Irish psychiatry. He returned to Ireland in 1963, where he worked at St Loman’s Hospital, before going on to serve as the director of the mental health section of the Irish Medico-Social Research Board (later Health Research Board) from 1969 to 2003 and the government inspector of Irish mental hospitals from 1987 to 2003. While many of his cultural explanations for Irish excessive drinking might be dismissed as merely speculative, his musings were, in fact, reflective of the broader research climate at that time.

From at least the 1930s, psychiatrists and other researchers had begun documenting the prevalence of Irish alcohol-related psychiatric admissions in the United States. Viewed alongside sociological studies that reinforced the enduring ‘drunken Irish’ stereotype, these findings seemed to suggest that the Irish, as a race, had an especially remarkable relationship with drink. By the 1960s, the alleged Irish susceptibility to alcoholism gained increasing attention from researchers and eventually officials in Ireland itself and continued to preoccupy commentators in the decades that followed. Significantly, this renewed awareness coincided with a shift in Ireland’s place on the international landscape, and was deeply intertwined with the broader social, cultural and political environment. While anxieties about the apparently rising incidence of alcoholism and alcohol-related harm were not unique to Ireland,^2^ the specific cultural meanings attached to excessive drinking in a nation internationally renowned for this problem mapped onto shifting international frameworks, informing medical perceptions and shaping policy.

This article explores expert and official interpretations of alcoholism and the ‘drunken Irish’ stereotype from 1945 to 1975. This period witnessed a number of important developments in the field including the introduction of the Irish Mental Treatment Act of 1945, the establishment of the Irish National Council on Alcoholism in 1966 and the creation of specialist alcohol treatment facilities in several psychiatric hospitals, many of which still operate today. This era also saw the adoption of the disease concept of alcoholism and later, the embracing by some actors of a public health perspective on alcohol. As will be argued, these contexts for understanding problem drinking were coloured by concerns that Irish social and cultural factors were contributing to rising levels of alcohol consumption and psychiatric admissions for alcoholism.

## Origins of the ‘drunken Irish’ stereotype

As Walsh observed in 1962, cultural associations between excessive drinking and the Irish date from over two millennia, when Plato noted the Celts as being ‘drunken and combative’.^3^ According to Elizabeth Malcolm, in her history of Irish temperance, from the sixteenth-century critical depictions of Irish drinking habits were routinely used to justify the attempted colonisation of an ‘uncivilised’ people.^4^ By the 1820s, Protestant landlords, clergy, doctors, lawyers and merchants had become considerably alarmed by the high levels of spirit consumption in Ireland. When viewed alongside Daniel O’Connell’s appeals to the Catholic masses in support of Emancipation, the spectre of the 1798 rebellion and the drunken violence perpetrated by both sides of the conflict loomed large.^5^ This apprehension led to the setting up by the Protestant middle classes of various temperance organisations throughout that century, the primary goal seemingly being to bolster their position in society while denigrating the customs of their Catholic social inferiors.^6^ Meanwhile, two notable Catholic temperance efforts were launched. The first was by a Capuchin friar, Fr Theobald Mathew, in 1838. Fr Mathew’s ‘crusade’ was to achieve astonishing, albeit short-lived success. From the time it was founded until roughly the beginning of the Great Famine in 1845, he is estimated to have single-handedly administered the abstinence pledge to between 700 000 and 2 million people.^7^ The second Catholic temperance endeavour, begun in 1898 by Fr James Cullen, was more enduring. The Pioneers Total Abstinence Association, still operating today, went from strength to strength in the first half of the twentieth century and, by 1959, claimed a membership of nearly 500 000.^8^ Yet, as Diarmaid Ferriter has shown, despite strong temperance activity and relatively moderate per capita consumption figures in the late nineteenth and early twentieth centuries, there remained a widespread belief that Ireland was ‘shaming itself, morally, politically and culturally by its heavy drinking’.^9^ For some increasingly militant, Irish nationalists in these decades, sobriety was therefore viewed as a key weapon for achieving political independence from Britain, an idea neatly summed up by the popular slogan, ‘Ireland sober, Ireland free’.^10^

These interpretations of Irish drinking were not lost on medical commentators in Ireland. While largely subscribing to broader European concepts of inebriety as a hereditary disease, practitioners were also influenced by specifically Irish social, cultural and political factors and their own political and religious leanings.^11^ By 1900, ‘intemperance in drink’ accounted for one in ten asylum admissions.^12^ Yet, in spite of the steady influx of alcohol-related cases, Irish asylum doctors were hesitant to claim expertise in the relatively newly defined and apparently incurable disease of inebriety.^13^ Moreover, in 1910, the long-held association between drunkenness and degeneration was seriously undermined, when a study by two scientists at the Galton Laboratory for National Eugenics at University College London concluded there was ‘no discernible connection between parental alcoholism and mental defects in their children’.^14^ By 1912, the Irish asylum inspectors had reached the same conclusion.^15^ The closure of the short-lived inebriate homes both in Ireland and Britain further weakened medical authority, given the very public support for this initiative by key players, including Dr Norman Kerr in the British context and Dr Ephraim MacDowel Cosgrave in Ireland.^16^ While discussion of the physiological and psychological effects of drinking persisted in Britain during the First World War,^17^ and America was making strides towards national prohibition, medical involvement in the Irish ‘drink question’ continued to wane during the 1920s. By the end of the First World War, alcohol had also come to assume far less importance for British and American medical commentators.^18^

That is not to say that the ‘drink question’ disappeared from the Irish political agenda. Following the War of Independence (1919–21) and the subsequent partition of the island into two separate states (1922–3), the Irish Free State government lost little time in reorganising liquor regulations. Coming under increasing pressure to tackle the licensed vintners, restrict pub opening hours and decrease the number of pub licences, the Cumann na nGaedheal party, who governed the Free State until 1932, responded through an intoxicating liquor commission and licensing acts between 1924 and 1927.^19^ Despite this government activity, alcohol did not re-emerge as a medical concern during this period. While this partly reflects declining medical interest in the ‘drink question’ during the interwar years internationally, anxieties about immunisation, coupled with persistently high infant mortality rates and infectious diseases, clearly took precedence in Irish medical discourses at this time. This period also reportedly witnessed a ‘general marked decrease’ in alcohol consumption in Ireland, while there was an estimated drop of one-fifth of reported deaths from liver cirrhosis between 1908 and 1949, in line with general trends in the United Kingdom.^20^

The lull in alcoholism research endured in Ireland until the 1960s when, as will be discussed, experts from various disciplines began publishing vigorously on the topic. Across the Atlantic, however, American psychiatrists and sociologists frequently cited high levels of drunkenness and alcoholism among Irish immigrants. These studies emanated predominantly from admissions data at mental hospitals in areas with large Irish-born populations, especially New York and Boston. For example, in 1930, Benjamin Malzberg, a psychiatrist and epidemiologist at the Statistical Bureau of the New York State Department of Mental Hygiene, offered several explanations for high rates of Irish alcohol-related admissions to New York State mental hospitals. These included the impact of migration, resultant cultural transplantation and persistence of social habits acquired in Ireland during the extreme hardship of the nineteenth century.^21^ A few years later, the head psychiatrist at the Johns Hopkins Hospital in Baltimore, Maryland, Dr Adolph Meyer, made several retrospective studies of alcoholic admissions to Massachusetts and New York State hospitals, which again demonstrated disproportionate rates among the Irish.^22^ Further evidence of a perceived Irish proclivity to alcoholism emerged in 1944, when Major Robert W. Hyde and Staff Sergeant Roderick M. Chisholm of Boston revealed the rejection rate for enlistees in the US army due to chronic alcoholism was higher in the Irish than for any other national group.^23^ Ten years later, a professor of social psychiatry, Bertham H. Roberts and professor of sociology, Jerome K. Myers at Yale University completed a prevalence study in New Haven. The results of this research indicated a far higher proportion of the Irish under treatment for alcoholism and drug addiction than other national groups.^24^ By now, there seemed little doubt among American researchers that there was something socially and culturally distinctive about Irish drinking behaviour.^25^

Harvard University sociologist, Robert F. Bales, certainly seemed to think so. Perhaps, the best-known twentieth-century writer on Irish drinking habits, in 1944, Bales completed his doctoral dissertation on the ‘Fixation Factor in Alcohol Addiction’, comparing Irish and Jewish social norms and attitudes towards drink.^26^ As Tanya M. Cassidy has noted, Bales apparently accepted the pervasive notion of an Irish predisposition to alcohol-related problems, in spite of ‘puzzling’ statistical evidence of low death rates from chronic alcoholism. In fact, she continues, Bales neither presented this data nor discussed the possible persistence of an unchecked stereotype in relation to Irish drinking behaviour.^27^ From 1962, Bales’ work attracted heightened recognition, after he published a shortened version of one of his dissertation chapters.^28^ Despite being cited widely as an authority on the topic thereafter, in reality, this publication was an already outdated and, at times, tentative portrait of Irish social life, based heavily on texts dating from the sixteenth to nineteenth centuries and containing no reference to research published after 1940. Despite these shortcomings, Bales’ work illustrated and reinforced several reasons popularly assigned to excessive drinking among the Irish.

According to Bales, drink, for the Irishman in the eighteenth and nineteenth centuries and now, it seemed, the 1960s, could serve as a medicine for almost any ailment, an ‘antidote to the symptoms of a hangover’, a preventive of damp and cold in the changeable Irish weather and especially for Irish working men who exhibited ‘a prevailing neglect or lack of proper clothing’, a food substitute in periods of famine or during religious fasts; and a reliever of sexual tension or even a ‘sexual substitute’ for young rural men in a society where marriage tended to favour only the first-born son.^29^ Moreover, drinking, Bales asserted, was ‘inextricably tied up with the expression of aggression in the Irish culture … most importantly, overt and active and persistent aggression against the English’. Consuming alcohol was not condemned, he continued, except where the family cash resources, or land tenure, were under threat. The practice of ‘treating’ or buying rounds for one’s companions or business associates only served to exacerbate the heavy drinking culture, and alcoholic beverages were omnipresent at christenings, funerals and wakes.^30^

In rendering his account of Irish life, Bales borrowed heavily from the work of two Harvard University colleagues, Conrad M. Arensberg and Solon T. Kimball, which contributed largely to the image of the mother-dominated Irish family. In 1937, these two anthropologists had settled in a rural community in Co. Clare in the West of Ireland where they observed and recorded typical rural Irish life. The resultant book, first published in 1940, presented some remarkably durable depictions and a second edition was published in 1968.^31^ In particular, the premise that heavy drinking among rural males was an important ‘safety valve in the release of sexual tension’ was to become a major theme for commentators in the late 1960s and 1970s.^32^ As will be discussed, Bales, too, remained highly influential.

The impetus for these studies might be attributed to an extension of anti-Irish prejudice in the wake of large-scale Irish migration to Britain and America during and after the Great Famine. Nineteenth-century popular attitudes connecting Irishness to the idea of ‘defectiveness’ had been propelled by high rates of mental disorders and confinement in asylums among Irish migrants.^33^ While by the twentieth century, Irish migrants to the United States had ‘left behind much of the discrimination and impoverishment’ they had previously endured, especially following the influx of ‘new immigrants’ from southern and eastern Europe, the hard-drinking Irish stereotype remained in the popular American imagination.^34^ Similarly, while the Irish in post-war Britain attracted relatively less negative political attention, due largely to the arrival of colonial immigrant populations from the Caribbean, India and Pakistan, older engrained anti-Irish sentiment lingered well into the 1960s and beyond.^35^

In addition to having large Irish immigrant populations, America was the birthplace of the new ‘disease view’ of alcoholism. This concept marked a departure from the nineteenth-century disease concept of inebriety, the key difference being the perception of drink itself. While previously alcohol had been portrayed as an inherently addictive substance, posing the risk that anyone who drank might lose control over their habit, the post-Prohibition perspective depicted it as a harmless substance for most, while the disease of alcoholism would descend on only a minority of vulnerable or ‘defective’ individuals.^36^ This framework filtered into the United Kingdom via E.M. Jellinek, a prominent epidemiologist and leader of the alcohol research programme at the World Health Organisation (WHO). Widespread acceptance of the disease view by the 1950s was also propelled by the spread of Alcoholics Anonymous (AA) from America to Europe.^37^

The transmission of American thought to the United Kingdom is further evidenced in English-based research on alcoholism in Irish immigrants. In 1956, two psychiatrists at Warlingham Park Hospital in Surrey, J.D. Sullivan and Max Meier Glatt, published the results of their investigation of Irish admissions to the institution’s alcoholic unit. While Sullivan and Glatt found the patients’ ‘Irish cultural background’ did not give rise to any significant differences in personality type, intelligence, age, sex or social status, they were struck by the fact that Irish Catholics who had lapsed in their faith tended to renew their religious practice during treatment and after discharge. They thus drew parallels with Italian Catholic psychiatric patients in the United States, where psychiatrists had established a relationship between decreasing intoxication and more frequent religious participation. In their discussion of alcoholism in Ireland, Sullivan and Glatt remarked there were no reliable figures. Instead, they relied on the formula devised by Jellinek, which held that between 5% and 6% of drinkers in any country were alcoholics. Combining this figure with observations about the institutionalised Irish abroad, the two psychiatrists arrived at the rather dubious-sounding estimate of between 23 000 and 75 000 alcoholics in Ireland. They therefore posited that ‘the problem of alcoholism among Irish people, living both at home and abroad, may be of some magnitude’.^38^

## State responses to alcoholism in Ireland, 1945–1968

By the mid-1950s, Irish commentators had begun responding to these American and English studies, both in the national press and in Irish medical journals*.* Partly in reaction to Sullivan and Glatt’s findings, which were synopsised in the *Irish Times* in November 1956, the medical correspondent for that newspaper summarised the dichotomy between domestic and international perspectives on Irish drinking habits:

We Irish have the reputation of being a great race of drinkers. Among ourselves the word ‘great’ in this context has a romantic ring about it, standing for Homeric or, perhaps, gargantuan. It suggests drinking long and deep, by big men with broad shoulders and hairy hands: quaffing by men who are exalted by drink, but who never become liquor’s slaves. To people of other lands, it merely means that we drink too much. Ethnically we stand out as drinking more than the Americans among whom so many of our brothers settle, and, if the English ‘popular’ Sunday newspapers are to be believed, the London-Irish are gamely doing their bit to maintain our name for turning out drinking-men that are second to none.^39^

The correspondent went on to lament that ‘you cannot have great drinkers without incurring a certain proportion of chronic alcoholics’, warning that this condition had become a ‘grave problem’ in Ireland.^40^

Despite growing recognition of alcoholism in Ireland, the lack of reliable data on the condition plagued contemporary analysts. Branding it the ‘skeleton in our cupboard’, the *Irish Times*’ medical correspondent decried the dearth of official figures, pointing out that statistics for several other European countries were available through the WHO. In a criticism that would later become commonplace, the author went so far as to suggest a wilful reluctance on the part of the Irish government:

One wonders if the authorities have deliberately refrained from finding out how many alcoholics there are in this country, lest the figures show us up in a bad light. Once compiled, the figures would have to be published for the information of other countries which are themselves dealing with the disorder. Is it that to let others know the true figures would be bad for ‘public relations’? Or is it that we are still trying to convince ourselves that there is no alcohol problem in Ireland, and therefore that it would be a waste of time looking for it?^41^

Whether owing to concealment, denial or even apathy, the fact remained that no attempt had been made to record alcoholism incidence levels since the creation of the Irish Free State in 1922. Prior to independence, alcohol had ranked high among causes assigned to asylum admissions. In 1919, drink was a factor in the admission of 495 (24.0%) men and 92 (6.1%) women to public asylums and 27 (16.7%) men and 11 (4.8%) women admitted to voluntary and private asylums.^42^ In 1909, 132 (6.7%) men and 43 (2.4%) women admitted to district asylums and 9 (6.8%) men and 2 (1.5%) women admitted to private and voluntary institutions were diagnosed with mania *a potu.* ^43^ From then until 1958, the inspectors of Irish mental hospitals did not enumerate patients’ aetiology or diagnosis. However, it is likely that high rates of alcohol-related psychiatric admissions continued during the early decades of the Irish Free State, which was characterised by a persistently heavy drinking culture.

Further evidence of the sustained role the Irish psychiatric services played in alcoholism treatment can be gleaned from the inclusion of ‘addict’ clauses in the Mental Treatment Act of 1945. Among its numerous aims, this Act sought to reduce patient numbers along with the stigma associated with mental illness, and introduced outpatient clinics and voluntary and temporary admission status. Ireland was not alone in pioneering innovative mental health legislation in this era. England and Wales, Northern Ireland and the US introduced equivalent laws in the 1930s and 1940s, providing for voluntary admission status.^44^ What marked the Irish statute out, however, was its explicit mention of addiction, which according to contemporary medical observers was a ‘unique feature’.^45^ The Act defined an addict as a person who, due to their addiction to drugs or intoxicants, was either dangerous to themselves or others, incapable of managing their affairs or of ‘ordinary proper conduct’ or was in serious danger of developing a mental disorder.^46^ As Shane Butler has deduced, in the context of mid-1940s Ireland, one can assume this definition referred principally to those deemed addicted to alcohol.^47^ This suggestion is supported by the examination of contemporary psychiatric records.^48^ Under the 1945 Act, an addict could be detained against their will as a ‘temporary patient’ for a period of up to 6 months.^49^ Applications for admission were preferably made by a spouse or relative, and accompanied by medical certification attesting to their need for at least 6 months’ psychiatric treatment.^50^ The ultimate decision then fell to the institution’s chief medical officer, who chose whether or not to receive the patient.^51^ If the patient was admitted, the chief medical officer assumed the authority to detain the patient for 6 months, release them prior to this period or if the patient escaped, to ‘retake’ them within 28 days.^52^ If the chief medical officer determined the patient would not recover within 6 months, they could extend the overall detention period by up to 2 years, with permission from the Minister for Local Government and Public Health.^53^

The Mental Treatment Act provided the first statutory guidelines for alcoholism treatment. A plausible reason for the focus on addiction in Ireland was a sustained influx of alcohol-related admissions combined with heavy drinking among the general public. Unlike in the United Kingdom, where levels of alcohol consumption had begun to fall significantly from the last quarter of the nineteenth century and remained low into the 1960s, post-war Ireland saw increased consumption of drink. This was partly because, due to Ireland’s neutrality, the state had not introduced wartime measures to curb beer production and promote ‘dry clubs’ as had been done by governments elsewhere.^54^ The focus on ‘addicts’ in the Mental Treatment Act might therefore be read as an indication that the Irish psychiatric profession, in concert with the government, had come to accept the new disease view of alcoholism by singling out alcoholics as a group in need of treatment. Yet, as Butler has persuasively argued, the background to the 1945 Act suggests that state support for this measure had been ‘tentative and equivocal’.^55^ In fact, the addiction clauses had been introduced almost as an afterthought following the Irish Medical Association’s intervention, and consultation between the Royal Medico-Psychological Association and the Department of Local Government and Public Health during the later stages of the legislative process.^56^

Despite this engagement between medical and state interests, the parliamentary secretary to that Department, F.C. Ward, underscored a general ambivalence when he asserted his reluctance to see mental hospitals routinely used as treatment centres for alcoholism except in cases of emergency. Ward instead urged the creation of separate specialist institutions.^57^ This was by no means a novel proposal. Some members of the medical profession had been suggesting this for more than half a century. Echoing the recommendations of Kerr and others in the late nineteenth century, in 1937, the author of a review article in the *Irish Journal of Medical Science* suggested that the best hope of curing an ‘addict’ would be in a ‘suitable institution’, one that was small, with extensive grounds and confined to members of one sex.^58^ This proposal was foreshadowed by the Mental Treatment Act, which permitted the government to order mental hospital authorities to provide ‘specified accommodation’ for temporary patients.^59^ It would, however, be another 20 years before this provision was availed of. In the meantime, and however unintentionally, general psychiatric facilities remained primary treatment sites for alcoholism.

Another rationalisation for the lack of government data on alcoholism might lie, to some extent, in the generally permissive attitude towards drunkenness in Irish society. A high level of alcohol consumption continued unabated during the 1950s and, as Ferriter has discussed, the prevailing ‘culture of toleration’ in this period meant excessive drinking was seen as both gregarious and entirely respectable.^60^ In addition, it emerged in that decade that the Irish were consuming less drink than their British neighbours. According to the recently established Central Statistics Office, in 1958, Ireland’s per capita consumption was 64.3 litres of beer and 1.2 litres of spirits, compared to the British figures of 79.1 and 1.1, respectively.^61^ Thus, in 1957, a State Commission on Intoxicating Liquor concluded drink was no longer a serious problem in Ireland. Two years later, when the Department of Justice queried the Department of Health about the scale of the issue of alcoholism, the latter estimated that less than 400 cases were admitted to institutions for treatment in any year.^62^ The Department of Health’s figure was proven incorrect the following year when its own inspector of mental hospitals reported that 561 patients (5.0%) admitted to Irish mental hospitals in 1958 had been diagnosed with ‘alcoholism’ and a further 92 (0.7%) with ‘alcoholic psychosis’.^63^

By the 1960s, the Irish reputation for drunkenness abroad was being openly renounced by the Irish government. In 1960, the country’s Taoiseach (head of government), Sean Lemass, complained of the ‘persistent and irritating falsehoods’ about the ‘drunken Irish’, stating that ‘even the BBC Television service rarely, if ever, presents a play about Ireland without the characters moving around in clouds of alcoholic vapour’.^64^ Importantly, Lemass was also angling for membership in the European Economic Community around this time and, in 1962, made a submission to Brussels to consider Ireland’s case for entry. This was, therefore, a period of transition for Ireland. From remaining neutral during the Second World War, and still quite conservative and insular during the 1950s, by the 1960s Ireland was becoming a more modern and outward-looking nation. It is, therefore, plausible that international perceptions of the Irish took on a renewed importance. This argument was summed up neatly by contemporary journalists*.* In 1964, Michael Viney, in the first of his series of *Irish Times* articles on ‘Alcoholism in Ireland’ reasoned:

Few Irishmen would disagree that the image of the ‘drunken Paddy’ whether fictionally presented on British television screens or factually endorsed in the magistrates’ courts of West London, is humiliating to the Irish at home. And if Mr Lemass is also concerned lest foreign industries are put off coming to Ireland through fears of drunken labour and absenteeism, this too is understandable.^65^

One Irish politician who *was* willing to confront the issue was George Colley, TD (member of Irish parliament). Speaking at a conference of his political party, Fianna Fáil, in January 1962, Colley proposed a campaign to eradicate alcoholism, including the setting up of alcoholic treatment units. In response to Colley’s statements, the Minister for Health, Sean McEntee, said that the matter was already being considered by the recently established Commission of Inquiry on Mental Illness. McEntee premised that alcoholism was ‘not such a grave problem’ in Ireland, that it was already being dealt with in mental hospitals with fairly satisfactory results and insisted he would have to wait for the Commissioners’ report before taking any action.^66^ A few months later, Colley reiterated his points in the Dáil (lower house of Irish parliament). Demonstrating an in-depth grounding in the literature on the topic, he applied the Jellinek formula to estimate that there were 50 000 alcoholics in Ireland, advising their treatment be looked into as a matter of urgency. While acknowledging the on-going investigations by the Commission on Mental Illness, he contended:

This whole concept of alcoholism as a mental disease is one of the causes of our failure to deal with the problem. If you have an alcoholic in your family, you will not advertise it. Apart from the ordinary disgrace involved in it, if it is officially treated as a mental disease, you want to avoid that stigma, if at all possible. The result is that there are many doctors who, for the sake of the patient or the patient's family, certify people as suffering from bronchitis and all sorts of diseases but never mention alcoholism, which they know is the real trouble.^67^

He therefore proposed the establishment of inpatient and outpatient facilities attached to general hospitals and urged the government to embark on an ‘intensive educational campaign’ led by the Department of Health.^68^

In spite of Colley’s exertions, the Department of Health seemed satisfied to wait for the Commission’s report. By the mid-1960s, however, there was mounting pressure on the government to at least pay lip service. In an editorial entitled ‘Action on Alcoholism’, an *Irish Times* correspondent, apparently unaware of Colley’s statements, decried the lack of state support:

In the past year or two, the public health problem of alcoholism has received unprecedented attention in medical, clerical and sociological circles, but not one deputy has spared time or interest to ask a relevant question in the Dáil … We have heard it suggested that to ask such questions in the Dáil would ‘do the country no good abroad’. This would be comically naïve were it not so monstrously cynical.^69^

The author went on to criticise the failure, in Ireland, to set up a national council on alcoholism along the lines of those operating in the United Kingdom and elsewhere, suggesting that past efforts had been ‘thwarted by the reluctance of public figures to have their names associated with the problem’.^70^ Government sensitivity towards the ‘drunken Irish’ stereotype was again alleged in the editorial, ‘Thirst for Truth’, in May 1965:

One suspects that the Government has been slow to give a lead in this matter through fear of appearing to confirm the ‘drunken Paddy’ image. If this were true, it would suggest a sad distortion of values. It is unlikely that Ireland produced vastly more alcoholism than any other country: and if it did, this would be all the more reason for acting preventively. There is a curious sensitivity in this country which makes the retort ‘We’re no worse than anywhere else’ a substitute for tackling the problem that does exist.^71^

The following day, it was announced that the Chief Justice, Cearbhall O’Dalaigh, had formed the Irish National Council on Alcoholism (INCA). Credit for this development was given to alcoholism experts at the two Dublin psychiatric institutions, St John of God Hospital and St Patrick’s Hospital, who had been advocating the establishment of such a body for several years.^72^ Both hospitals had developed specialist alcoholic treatment facilities in the 1960s and were widely held to be leading the way in this field. The immediate impetus for establishing INCA had arisen from a three-day International Symposium on Alcohol and Alcoholism held earlier that week at St John of God Hospital.^73^ Deputising for the Minister for Health, Colley, who was now the Minister for Education, gave the opening address, in which he underscored the lack of alcoholism statistics for Ireland but asserted the matter was of ‘sufficient proportions to merit the most serious consideration’.^74^ During the course of proceedings, speakers highlighted the need for a council on alcoholism to carry out preventive work, disseminate information about the disease view among the public to remove the stigma attached to alcoholism and promote research to discover the extent of the problem in Ireland.^75^

These activities were also among the recommendations laid out in the long-awaited report of the Commission of Inquiry on Mental Illness, which was published in 1966 and, as anticipated, included a section on ‘alcoholics’. The Commissioners, who both ‘welcomed and endorsed’ the Council’s formation, verified that the prevalence of alcoholism in Ireland was unknown. Predictably, this report was less accusatory in tone than the contemporary press. By way of explanation, it drew attention to the absence of common standards in diagnosis, the degree of social concealment and the treatment of symptoms in isolation which, it asserted, ‘all help to obscure the epidemiological picture’. The Commissioners went on to report they could find no detailed survey of the number of alcoholics in Ireland but were aware of American and British research, which appeared to show that ‘Irish emigrants, or people of Irish descent, occupy a high place when alcoholics are classified by country of origin or by ethnic group’. However, they contested the premise that such findings indicated the extent of alcoholism in Ireland, stating that ‘emigrants and their immediate dependants are frequently subjected to exceptional social pressures conducive to heavy drinking’.^76^ Despite uncertainty surrounding the prevalence of alcoholism in Ireland, the Commissioners declared themselves satisfied, based on the experience of practising Irish psychiatrists, that the problem was both serious and required immediate attention.^77^ They therefore recommended that sample surveys of Irish drinking patterns and the probable prevalence of alcoholism be undertaken through co-operation between the Medico-Social Research Board, the Medical Research Council, INCA and ‘other interested voluntary bodies’, with a reasonable proportion of the cost to be borne by the State.^78^

By 1967, INCA had begun operating and received an initial grant of £2,000 from the Department of Health.^79^ The driving force behind the Council, however, was a handful of private sector psychiatrists together with selected members of AA, which had established its first European base in Dublin in 1946.^80^ The organisation’s first executive director was Richard Perceval, previously the director of the UK’s National Council on Alcoholism and a former St Patrick’s patient and AA member.^81^ Within 2 years, INCA had reportedly dealt with 561 cases of alcoholism and was sponsoring a £30 000 survey of the incidence, causes and effects of alcoholism in Ireland. The information obtained was to be presented to the Department of Health to inform the implementation of a national policy. Alongside this, there were plans to develop information, education and advisory services.^82^ Commenting on the progress of the INCA-sponsored survey in November 1968, O’Dalaigh was pleased to announce that it had already attracted international interest:

Ireland can now be regarded as playing a part in the work of investigating alcoholism and of rehabilitating not only alcoholics but also their families. Although a very great deal remains to be done, I think there can be justifiable pride in what has been achieved so far.^83^

Following these developments, Irish officials were apparently more comfortable with the prospect of openly promoting and sponsoring alcoholism research. In December 1969, the new Minister for Health, Erskine Childers announced: ‘the problem of alcoholism has been swept under the carpet for a long time and next year I am going to take it from under the carpet when I have considered measures to deal with it’.^84^ True to his word, albeit with some delay, in November 1971, Childers requested INCA to submit a detailed report to his department, examining the problem of alcoholism, seeking information, evidence and views from other organisations and individuals engaged or interested in the alcoholism field, recommending priorities for practical action and setting out the financial implications of such steps.^85^ This request was satisfied in 1973, when INCA presented its first major report. As will be discussed, this document drew on the growing body of Irish-based research into alcoholism, which by then had begun in earnest.

## Interpreting alcoholism in Ireland, 1968–1975

By the late 1960s, assessments of the Irish predisposition to alcoholism abroad, whether based on genetic or psychological makeup, or some inherent social or cultural flaw, began to preoccupy researchers in Ireland. Much of this discussion took place in the pages of the *Journal for the Irish Medical Association*, which boasted strong participation from disciplines including psychiatry, psychology and pharmacology. This research activity was initially led by Dermot Walsh. In his 1962 article, Walsh had proclaimed all authorities unanimous that Irish rates of alcoholism were among the highest of any national group.^86^ Like Sullivan and Glatt’s Warlingham Park study, however, Walsh based his findings on psychiatric admissions outside of Ireland. Mindful of potential divergences among the Irish in Ireland, he conceded that high rates of alcohol-related admissions among emigrants might be explained by the fact that those on a ‘short drinking bout’ were more conspicuous in a culture with less tolerance for drunkenness. In other words, he quipped, ‘there is a world of a difference in effect and consequence between getting drunk on a Saturday night in rural Ireland and doing the same thing in urban New York’.^87^

On his return to Ireland, Walsh began conducting intensive domestic surveys of alcoholism. His investigation of first admissions to Dublin psychiatric facilities during 1962 was published by the *JIMA* in 1968*.* In a statement that would prove highly provocative, this article concluded that alcoholism was a ‘major public health problem in Dublin’, accounting for at least one-quarter of all first admissions to psychiatric hospitals. While Walsh acknowledged that most of the evidence to date was impressionistic, he suggested his Dublin study, which found a first admission rate of 34.4 per 100 000 population aged 10 and over, confirmed the ‘American findings’ so often cited in the past. Walsh calculated that Dublin’s male first admission rate was twice as great as the Scots and twelve times that of England and Wales, while the female rate was eight times that in England and Wales.^88^ The psychiatrist also drew attention to a separate analysis he had recently conducted, confirming his main findings for Dublin at a national level. Unsurprisingly, given mounting public demands for such evidence, this Irish-based research reached a wide audience. In 1969, an article based on Walsh’s nationwide survey was published in the *British Journal of Psychiatry* (*BJP*) and its key findings were subsequently summarised in the *Irish Times.* ^89^

Walsh’s very public and in the case of the *BJP* article, international, assertion that the Irish in Ireland were disproportionately prone to alcoholism created waves among other workers. In 1970, a professor of psychology and researcher at the Irish Economic and Social Research Institute, Richard Lynn,^90^ with his assistant Susan Hampson, responded with a comprehensive rebuttal in the *JIMA.* They argued that compared to the United Kingdom, Europe and the United States, Ireland had a lower per capita drink consumption, lower death rates from alcoholism and liver cirrhosis and lower conviction rates for drunkenness. They also laid waste to suggestions that high incidence of alcoholism among Irish immigrants in the United States signified high prevalence in Ireland itself.^91^

The academic squabble continued when Walsh countered with a letter to the *JIMA*’s editor, reaffirming alcoholism as ‘an important national health issue’. On the topic of per capita drink consumption, Walsh made a valid observation by highlighting the figures’ failure to consider the high proportion of total abstainers in Ireland.^92^ This point had also been raised in the report of the Commission on Mental Illness in 1966 and held some weight.^93^ The popularity of temperance in Ireland had reached something of a climax in the years leading up to Walsh’s nationwide study, which had been conducted in 1964. In 1959, the Pioneers Total Abstinence Association claimed to have nearly 500 000 members registered in the 1 900 centres around the country and over 90 000 had attended the Association’s Diamond Jubilee celebration in Dublin’s Croke Park stadium that year.^94^ These figures are rendered even more remarkable in the context of a population of less than 3 million. Walsh also took issue with Lynn and Hampson’s lower death rates argument, pointing out that alcoholism was not necessarily a fatal disease, while the relationship between alcoholism and liver cirrhosis remained uncertain. On conviction rates for drunkenness, he suggested these statistics might be more illustrative of societal attitudes rather than incidence of alcoholism. Finally, he concluded that first admission rates for alcoholism in Dublin, Ireland and for the American Irish were similar, consistent and pointed unequivocally to the conclusion that ‘for the Irish, wherever they may be, alcoholism is a serious problem’.^95^

The debate did not rest there. Beneath Walsh’s letter, the editor published a reply from Lynn and Hampson. The two researchers insisted their methodology was generally accepted by workers in the field as being indicative of prevalence rates. Moreover, they contended, ‘if the results given in his paper and ours are taken together, it appears that the Irish rates are lower than those of France, Canada, Helsinki, and the Virgin Islands and the only country with a lower rate than Ireland is Britain’.^96^ These exchanges were symptomatic of the kind of professional disagreement about not just the scientific nature or cause of alcoholism, but its prevalence. It also hinted at a level of national sensitivity, if not bias, among certain experts, with Lynn and Hampson asserting ‘the object of our paper was to question the beliefs that the Irish are heavy drinkers’.^97^

By now, leading pharmacologists had also entered the debate. In January 1968, Cedric William Malcolm Wilson, a Edinburgh-born and educated Professor of Pharmacology at Trinity College Dublin delivered a paper to the Royal College of General Practitioners on the social implications of drug use. In it, he asked whether the Irish possessed a ‘specific genetic or cultural predisposition for alcohol’ and described investigations on-going at Trinity to determine whether a taste for drink could be defined objectively or altered pharmacologically. If, as Wilson seemed to suspect, ‘the taste for drink is a characteristic of the Irish’, this scientific research might form the basis for measuring it and even controlling it with drugs. He went on to issue a call to arms:

It seems that the time has come to combine the scientific, clinical and sociological expertise in the country to find out if it is the Irish culture which causes the Irishman to drink, of if it is the Irishman’s genes which causes the American to get drunk.^98^

Notwithstanding disagreement over the cause of alcoholism, the increasing proliferation of Irish research on alcoholism reflected growing recognition of the disease view. Certainly, by the 1960s a number of Irish psychiatrists were openly advocating the theory. It also gained official approval in the report of the 1966 Commission on Mental Illness, which Butler has described as the ‘clearest and most unequivocal public policy acceptance of the disease concept’.^99^ Possibly its most avid individual proponent was John G. Cooney, a consultant psychiatrist at St Patrick’s who became one of Ireland’s leading authorities on the psychiatric treatment of alcoholism. Speaking at the North Dublin Medical Club Symposium in 1963, Cooney urged his medical colleagues to accept the disease view:

Too often doctors have allowed their view of alcoholics to be distorted by emotional factors. Commonly their own subconscious fears regarding alcoholism have been projected on to their alcoholic patients. If one is to treat alcoholism successfully whether in hospital of in general practice one must feel as well as believe that the alcoholic is ill and suffering from a disease just as surely as a diabetic is suffering from his excess blood sugar.^100^

Cooney also advocated for the admission of alcoholic patients to hospitals, ‘preferably one which specialises in the treatment of alcoholism’.^101^ This is unsurprising, given the psychiatrist’s position in a semi-private psychiatric hospital with such facilities.^102^ Cooney was responsible for the establishment of a specialist treatment programme for alcohol-related disorders at St. Patrick’s, published extensively on the topic of alcoholism and was a founding member of INCA.^103^ Like his colleagues overseas, Cooney was therefore asserting psychiatry’s expertise in alcoholism treatment.^104^ Simultaneously, there was a marked rise in the number of alcohol-related admissions to psychiatric hospitals from 561 in 1958 to 1 964 in 1967.^105^ By this point, the role played by psychiatric services for alcoholism in Ireland had crystallised and psychiatrists had apparently grown more comfortable with this function.

The central tenet of the disease view, however, did not sit well with many Irish commentators. After all, the premise that alcoholism constituted an inherent ‘flaw’ in the individual was a difficult pill to swallow in a country with increasing psychiatric admissions for that very disorder. As has been demonstrated, political sensitivity towards the ‘drunken Irish’ stereotype had delayed state responses to alcoholism. Concerns surrounding Irish national identity, especially on the international stage, were only compounded by the suggestion that a large percentage of the Irish populace, whether at home or abroad, were somehow ‘defective’. Illustrating this point in 1962, a consultant psychiatrist at St John of God Hospital, Dr Desmond McCarthy, complained:

One of the great difficulties in this country was that alcoholism was not accepted as an illness. It still carried a social stigma, a rather foolish way of looking at a serious disease. The basic illness was often hidden under other names for face-saving thus there were no reliable figures for alcoholism.^106^

Evidence of a persistent stigma around alcoholism among doctors was presented as late as 1969. Reporting on an alcoholism seminar for general practitioners in Waterford that May, the *Irish Times*’ medical correspondent, David Nowlan, wrote of the survival within the Irish medical profession of ‘medieval attitudes’. Nowlan described how one general practitioner had stood up at the end of the seminar and ‘stated quite categorically that alcoholism was a sin in the face of God and against God’s works deserving of only censure and moralistic indignation’.^107^

Members of the Irish public were also sensitive to the implications of the disease view. In 1972, Mrs S.J. Hanly, a frequent contributor of letters to the *Irish Times*, wrote in response to Walsh’s findings:

The rest of the scientific world claims they cannot find any one cause of alcoholism. It’s nice to know that we find just being Irish is the cause of our alcoholism … It would be verbal arrogance on my part to question Dr Walsh’s diagnosis or prognosis. I wouldn’t dare to do so. I just wonder if there is any way out of this ‘most susceptible to alcoholism’ trap, when I hadn’t the good fortune to have ancestors born in England, Scotland, Wales or Europe.^108^

Perhaps, it was partly due to this national sensitivity that the preferred explanation for the cause of alcoholism tended to be cultural rather than genetic. As discussed, Walsh had emphasised cultural influences on Irish alcoholism early on, in his 1962 article. Cooney also lent some weight to this interpretation. Speaking at a symposium at the Royal College of Physicians of Ireland in September 1971, Cooney sought to disentangle the socio-cultural and psychological reasons for alcoholism in the Irish. While, he surmised, it was tempting to view the condition as a type of ‘inborn error of metabolism’, he was quick to stress that the facts did not support this theory. Instead, Cooney supported a ‘multifactorial theory’ along the lines that exposure to a critical amount of alcohol in a favourable setting would predispose certain individuals by reasons of metabolic and/or personality factors, to develop the disease.^109^

Despite this rather ambiguous evaluation, the psychiatrist devoted some space to the impact of social and cultural shifts in Ireland. Modernisation, he proposed, had brought with it a variety of new factors that were now influencing Irish drinking habits. These included increasing social mobility in rural Ireland leading to more money being spent on drink; the replacement of dimly lit, all-male pubs with brightly lit bars and singing lounges catering to younger married couples; expense account drinking in the cities following the patterns of London and New York; and the centrality of alcohol on all social occasions and in many business transactions. Cooney’s observations were not unfounded, and indeed reflected contemporary anxieties about broader social and cultural change. The 1960s had been a decade of relative economic growth and modernisation for Ireland, one which witnessed rising living standards, a resulting marriage boom, falling emigration, increasing liberalisation of society, the arrival of television and foreign influences and the weakening of authority of the Catholic Church.^110^ Alongside more liberalised attitudes, especially among young adults, greater disposal income corresponded to a dramatic climb in expenditure on drink, with spirit consumption alone rising by 66% between 1963 and 1972.^111^ Meanwhile, official estimates of the ‘true’ number of alcoholics in Ireland were now within Sullivan and Glatt’s 23 000–75 000. Inevitably, Cooney argued, ‘all this exposure to alcohol has led, in the opinion of many workers in the field, to an increase in alcoholism’.^112^

Cooney’s concerns about rising exposure to alcohol were illustrative of those in Ireland and elsewhere. The 1970s marked a turning point in attitudes towards drinking and alcoholism across many countries. As Betsy Thom has shown, following the discovery by epidemiologists that rising per capita alcohol consumption in a society led to a concurrent increase in alcohol-related harm, drink came to be presented as a problem for ‘total populations’ rather than a predisposed minority. Designated the ‘public health’ perspective, this approach gradually supplanted the disease concept in the ensuing decades.^113^ Internationally, within this new paradigm, social contexts rather than psychological or biological causes of excessive drinking were underlined. In the Irish case, this led to a renewed examination of Irish cultural as well as social influences.

For Cooney, recent shifts in relation to religious devotion were especially pertinent:

The rapid advances in science, the challenge to authority, the reappraisal and questioning of traditional values and religious beliefs have all affected life here in Ireland. Inevitably this has led to a sense of perplexity and confusion in people’s minds as to what direction they are taking in their role in the changing world of today.^114^

This litany almost certainly referred to the impact of the recent Vatican II reforms within the Catholic Church, which were intended to significantly liberalise the practice and scope of Catholicism, including a questioning of church control in the realm of areas like education.^115^ His musings reflected those of many contemporaries who were anxious about the impact such changes would usher in, as opinions were divided among both the clergy and laity over several thorny issues, including social reform, censorship and sexual behaviour.^116^ The doctor, himself, was a man of ‘strong faith’, who spent most of his career working closely with the Daughters of Charity.^117^

Others, however, cast aspersions on the conservative Catholicism still dominating many areas of Irish cultural and social life, arguing that its stifling nature was a catalyst for excessive drinking, and could even lead to alcoholism. A number of these concerns were outlined by the journalist, Mary McCutchan, in her series of *Irish Examiner* articles on ‘Alcoholism in Ireland’ in 1968. One issue identified was the Confirmation pledge taken by most Catholic children in Ireland at the age of eleven or twelve, who promised to abstain from alcohol until the age of twenty-one or twenty-five. This practice, according to some researchers, could result in a ‘false and unnatural attitude towards alcohol’.^118^ The strict celibacy rules the Catholic Church imposed on unmarried people were also singled out as a reason for excessive drinking to relieve sexual frustration. In addition, it was argued, a ‘substantial amount of sexual repression’ still existed in Ireland due to the tendency of Irish men to marry late and the unavailability of birth control, which caused many married couples to abstain for longish periods in order to limit their family size.^119^ Such interpretations of ‘uniquely’ Irish cultural influences largely echoed those of the American sociologists and other researchers outlined in section one. They also mapped on to broader debates about Irish culture more generally at a time when attempts at modernity and social and cultural change were limited by the continued influence of the Catholic Church in many areas. As Mary E. Daly has shown, marriage and sexual morality, in particular, had been major exceptions to this process of reform into the 1970s and beyond.^120^

An increased focus on socio-cultural factors was also visible in INCA’s report to the Minister for Health in 1973. As Butler has established, by this point internal tensions had developed within the Council between its broad membership of public and private sector psychiatrists, who were more aligned to the disease view, and other researchers and epidemiologists, who might be expected to promote the public health perspective. Perhaps, predictably, given its status as a council on alcoholism, the report placed explicit emphasis on the disease model. Nonetheless, it also gave some indication that the new public health approach was gaining hold.^121^ The language employed revealed recognition of the potentially harmful effects of heavy drinking, which were, it was stated, at least as dangerous as alcoholism and often more far-reaching in the social and economic context because of the greater numbers involved.^122^ The report therefore recommended, as a crucial step, the prevention of both alcoholism and excessive drinking by changing social and cultural attitudes towards alcohol.^123^

Allegedly inherent links between heavy drinking and Irish culture were also underscored by the Director of the Medico-Social Research Board, and general practitioner, Dr Geoffrey Dean. In a submission to INCA, which was retrospectively added to the report in November 1973, Dean premised that:

Because cultural factors appear to be predominant in determining drinking behaviour, we need much more understanding of the underlying motivation that determines the culture of drinking behaviour.^124^

Among the issues Dean identified were the ‘rather extreme attitude’ of the Catholic Church in its condemnation of sins involving sex, the authoritarian nature of the Irish education system and attendant pressures to conform to the cultural norm, and the loneliness created by the segregation of the sexes both in schools and within broader social activities. In contrast to Cooney’s concerns about young couples frequenting ‘brightly lit bars and singing lounges’, Dean welcomed ‘respectable’ women’s newly granted access to public houses. He hoped that men and women drinking together in a ‘more relaxed setting’ would result in ‘more reasonable drinking, particularly as the custom of standing rounds is becoming less of a rule’.^125^

In attempting to account for what especially about Irish culture might create a fertile ground for alcoholism, the discipline of sociology once again came to the forefront. This time, the leading investigator was a University College Dublin social scientist, Joyce Fitzpatrick (later O’Connor). In the early-1970s, Fitzpatrick carried out major surveys, investigating attitudes towards alcohol and drinking patterns among the Irish in Ireland, the first- and second-generation Irish in England and the English in England. Billed as the first European study of its kind, the project was based on empirical research, incorporating intensive interviews over a 3-year period with more than 2 100 youths aged 18–21 and their parents.^126^ In line with the recommendations of the 1966 Commission on Mental Illness, the work was jointly sponsored by the Irish Department of Health, INCA, the Medico-Social Research Board and later the Medical Council on Alcoholism of Great Britain.^127^ The findings were also published in book format in 1978.^128^

A central concern for Fitzpatrick was to interrogate the ‘drunken Irish’ stereotype. She was critical of the work of Bales’ and others, whom, she noted, had helped to reinforce this image of the Irish as far back as 1946. In her critique of Bales’ research, she argued that ‘some of his basic assumptions were faulty and his sources incomplete’.^129^ One area of difficulty lay with the fact that Bales had based his analysis of Irish life exclusively on the work of Arensberg and Kimball, which, as Fitzpatrick pointed out, had contributed largely to the image of the mother-dominated Irish family, as well as given rise to the idea of drink as a reliever of sexual tension among rural Irishmen.^130^ It is likely that Fitzpatrick was also covertly critiquing Walsh’s work, which, too, had highlighted the ‘peculiar’ nature of Irish family structure. As she noted, more recent studies had challenged this view of Irish family life.

Fitzpatrick’s research received broad coverage in the contemporary media and was heralded by many as finally satisfying appeals for a comparative Irish-based investigation of Irish drinking habits.^131^ While her work confirmed the ‘image of the Irishman in England as a heavy-type drinker’, she demonstrated that fewer Irish people drank than either the Irish in England or the English, giving rise to many a triumphant headline in the Irish national press. Moreover, Fitzpatrick argued that ethnicity was not a major factor, given that ethnic differences had not been found to persist from one generation to the next.^132^ Yet, Fitzpatrick did identify some areas of cultural difference, which created a confusing image of domestic Irish drinking behaviour. While confirming there was a higher proportion of abstainers in Irish society owing to the sustained influence of the Pioneers Total Abstinence Association, when it came to alcohol-related problems, the study revealed that Anglo-Irish and Irish groups fared worst, having more relatives or close friends with drink-related problems and Irish children being more likely to have experienced problems in their home life due to drink.^133^ Fitzpatrick’s work, therefore, presented statistical and qualitative evidence of a sustained ambivalence in the Irish relationship with drink, one which had long been hinted at by commentators attempting to challenge the notion of the Irish as a nation of hard-drinkers. As Ferriter has phrased it, Ireland in this period was presented as a ‘nation of extremes’.^134^

By now researchers and other workers in Ireland were apparently working together to openly challenge the ‘drunken Irish’ stereotype. In 1972, the new director of INCA, Joseph Adams, asserted: ‘I’ve been trying to explode the myth of the drunken ‘Paddy’ for some time’.^135^ In the same year, and just a decade after his ‘Cultural Influences’ article had been published, Walsh had also altered his tone. Commenting on developments in the alcoholism field over the previous two decades, Walsh wrote:

I think it is clear that these activities, whether therapeutic, research or preventive…indicate the concern with what is a characteristically Irish problem, the moving away of public attitudes from accepting a stereotype of Irish role-fulfilment towards a standpoint of responsible public attitudes to the problems of alcoholism.^136^

In addition, the Irish state was now openly embracing new methods to tackle both alcoholism and excessive drinking. In late 1973, the Department for Health sponsored a £50 000 anti-drink advertising campaign in newspapers and television, based on the recommendation of the INCA report.^137^ While part of the aim was to better educate the Irish public about the disease of alcoholism, there was also an emphasis on changing Irish attitudes to drink.^138^ The following year, the Minister for Health and Social Welfare, Brendan Corish, sanctioned a grant of £150 000 towards the construction of a new research and admissions unit at St. Patrick’s. Speaking at the announcement of this development, Corish expressed his hope that one of the benefits to flow from the new research unit would be fresh insights into the problem of alcoholism.^139^

## Conclusion

Much prominence has been given, in histories of problem drinking and alcoholism, to the adoption of the disease model and its subsequent displacement by the public health approach. While this paradigm applies readily to the Irish context, it is notable that twentieth-century discussions of alcoholism among the Irish began with international observations of immigrant populations in America and Britain. The result was the embedding of a sustained cultural stereotype of the Irish, whether at home or abroad, as being not just heavy drinkers but somehow predisposed to the disease of alcoholism.

In the 1950s, external expert commentary on Irish drinking behaviour began to gain publicity in Ireland, as evidenced in the national newspapers. By the 1960s, the Irish state was progressively called upon to respond to the issue of alcoholism. A central concern for critics was the lack of statistical information in Ireland, especially when viewed alongside the availability of international figures through the WHO’s promotion of epidemiological work and data sharing. Explanations for the government’s reluctance to take action on alcoholism included accusations of concealment or denial. Certainly, the implication that alcoholics were somehow genetically or psychologically ‘defective’ at a time when the Irish were believed to be disproportionately prone to this ‘defect’, did little for the national identity of a country whose international profile was becoming more important for its leaders. By the mid-1960s, however, increasing emphasis on the Irish ‘drink question’ led to the establishment of an Irish National Council on Alcoholism and the eventual sponsorship by the Department of Health of research, treatment facilities and preventive measures.

These events coincided with an upsurge in Irish-based research on alcoholism. The researchers responsible were not only influenced by the disease view and later public health frameworks, but also appeared to be deeply self-conscious about external commentary on Irish drinking behaviour. It could therefore be argued that while the disease view sat uneasily with those sensitive to negative international perceptions of the Irish, the public health perspective provided a welcome opportunity to tackle social and cultural issues, and, with them, the inherent stereotypes about drinking behaviour in Ireland.

## Footnotes

1

Dermot Walsh , ‘Cultural Influences in Psychiatric Illnesses in the Irish’, Journal of the Irish Medical Association, 50, 297 (March 1962), 62–8, on 65–6.14004730

2 See, for example, Stephen White , Russia Goes Dry: Alcohol, State and Society (New York: Cambridge University Press, 1996); Mat Savelli, ‘Diseased, Depraved or just Drunk?: The Psychiatric Panic over Alcoholism in Communist Yugoslavia’, *Social History of Medicine*, 25, 2 (2011), 462–80; Mark Lawrence Schrad, *Vodka Politics: Alcohol, Autocracy, and the Secret History of the Russian State* (Oxford: Oxford University Press, 2014); James Nicholls, *The Politics of Alcohol: A History of the Drink Question in England* (Manchester and New York: Manchester University Press, 2009); Virginia Berridge, *Demons: Our Changing Attitudes to Alcohol, Tobacco & Drugs* (Oxford: Oxford University Press, 2013).

3 Walsh, *op. cit.* (note 1), 66.

4

Elizabeth Malcolm , ‘Ireland Sober, Ireland Free’: Drink and Temperance in Nineteenth-Century Ireland (New York: Syracuse University Press, 1986), 3.

5 Malcolm, ‘*Ireland Sober, Ireland Free’*, 55–6.

6

Elizabeth Malcolm , ‘Temperance and Irish Nationalism’, in F.S.L. Lyons and R.A.J. Hawkins (eds), Ireland under the Union: Varieties of Tension (Essays in Honour of T.W. Moody) (Oxford: Clarendon Press, 1980), 69–114, on 113.

7

Colm Kerrigan , Father Mathew and the Irish Temperance Movement 1838–1949 (Cork: Cork University Press, 1992), 25–6; Paul A. Townsend, *Father Mathew, Temperance and Irish Identity* (Dublin: Irish Academic Press, 2002).

8

Diarmaid Ferriter , A Nation of Extremes: The Pioneers in Twentieth-Century Ireland (Dublin: Irish Academic Press, 1999), 191.

9

Diarmaid Ferriter , ‘Drink and Society in Twentieth-Century Ireland’, Proceedings of the Royal Irish Academy, 115C (2015), 1–21, on 4.

10 Malcolm, *op. cit.* (note 6), 111–5.

11

Alice Mauger , ‘“The Holy War Against Alcohol”: Alcoholism, Medicine and Psychiatry in Ireland, c. 1890–1921’, in Steven J. Taylor and Alice Brumby (eds), Healthy Minds in the Twentieth Century: In and Beyond the Asylum (Basingstoke: Palgrave MacMillan, 2019), 17–52.

12

Mark Finnane , Insanity and the Insane in Post-Famine Ireland (London: Croom Helm, 1981), 146.

13 See Mauger, *op. cit.* (note 11).

14 Berridge, *op. cit.* (note 2), 91.

15

Catherine Cox , Negotiating Insanity in the Southeast of Ireland, 1820–1900 (Manchester: Manchester University Press, 2012), 62.

16

Virginia Berridge , ‘The Origins and Early Years of the Society 1884–1899’, British Journal of Addiction 85 (1990), 991–1003 10.1111/j.1360-0443.1990.tb02605.x2245248; Mauger, *op. cit.* (note 11); Nicholls, *op. cit.* (note 2), 177.

17 Nicholls, *op. cit.* (note 2), 176.

18 Berridge, *op. cit.* (note 2), 144.

19 Ferriter, *op. cit.* (note 9), 5–6; Shane Butler , Benign Anarchy: Alcoholics Anonymous in Ireland (Dublin: Irish Academic Press, 2010), 24–8.

20 Ferriter, *op. cit.* (note 9), 9; Berridge, *op. cit.* (note 2), 165.

21 Benjamin Malzberg, ‘Mental Disease and “the Melting Pot”’, *Journal of Nervous and Mental Disease*, 72, 4 (1930), 379–95; Benjamin Malzberg, *Social and Biological Aspects of Mental Disease* (Utica, New York: State Hospitals Press, 1940).

22

Adolph Meyer , ‘Alcohol as a Psychiatric Problem’, in Haven Emerson (ed.), Alcohol and Man (New York: Macmillan, 1933), 296–7. Cited in Richard Stivers, *A Hair of the Dog: Irish Drinking and American Stereotype* (Pennsylvania: The Pennsylvania State University Press, 1976), 10.

23

Robert W. Hyde and Roderick M. Chisholm , ‘Studies in Medical Sociology: The Relation of Mental Disorders to Race and Nationality’, New England Journal of Medicine 231 (1944), 612–18.

24

Bertham H. Roberts and Jerome K. Myers , ‘Religion, National Origin, Immigration and Mental Illness’, American Journal of Psychiatry, 110, 10 (1954), 759–64.10.1176/ajp.110.10.75913138754

25 For further discussion of the research on Irish alcohol-related admissions to US mental hospitals, see Stivers, *op. cit.* (note 22), 5–14.

26 This 1944 dissertation was later published as Robert F. Bales , The “Fixation Factor” in Alcohol Addiction: An Hypothesis Derived from a Comparative Study of Irish and Jewish Social Norms (New York: Arno Press, 1980).

27

Tanya M. Cassidy , ‘Irish Drinking Worlds: A Socio-Cultural Reinterpretation of Ambivalence’, International Journal of Sociology and Social Policy, 16, 5 (1996), 5–25, on 7–8.

28

Robert E. Bales , ‘Attitudes towards Drinking in Irish Culture’, in David J. Pittman and Charles R. Snyder (eds) Society, Culture and Drinking Patterns (London and New York: John Wiley & Sons, 1962), 157–87.

29
*Ibid.*

30
*Ibid.*, 173.

31

Conrad M. Arensberg and Solon T. Kimball , Family and Community in Ireland (Cambridge, Mass: Harvard University Press, 1940); Conrad M. Arensberg and Solon T. Kimball, *Family and Community in Ireland*, 2nd edn (Cambridge, Mass: Harvard University Press, 1968).

32 Personal Communication, C.M. Arensberg, cited in Bales, ‘Attitudes towards Drinking in Irish Culture’, 168–9.

33 See, for example, Catherine Cox, Hilary Marland and Sarah York, ‘Emaciated, Exhausted and Excited: The Bodies and Minds of the Irish in Nineteenth-Century Lancashire Asylums’, *Journal of Social History*, 46 (2012), 500–24; Catherine Cox and Hilary Marland, ‘“A Burden on the County”: Madness, Institutions of Confinement and the Irish Patient in Victorian Lancashire’, *Social History of Medicine*, 28, 2 (2015), 263–87; Elizabeth Malcolm, ‘“A Most Miserable Looking Object” – The Irish in English Asylums, 1851–1901: Migration, Poverty and Prejudice’, in John Belchem and Klaus Tenfelde (eds), *Irish and Polish Migration in Comparative Perspective* (Essen: Klartext-Verlag, 2003), 121–32; Vishal Bhavsar and Dinesh Bhugra, ‘Bethlem’s Irish: Migration and Distress in Nineteenth-Century London’, *History of Psychiatry* 20 (2009), 184–98; J. W. Fox, ‘Irish Immigrants, Pauperism, and Insanity in 1854 Massachusetts’, *Social Science History* 15 (1991), 315–36.

34

Kevin Kenny , The American Irish: A History (London and New York: Longman, 2000), 199–202.

35 Enda Delaney, *The Irish in Post-War Britain* (Oxford: Oxford University Press, 2007), 75–6; 117; 122–6.

36

Harry G. Levine , ‘The Discovery of Addiction: Changing Conceptions of Habitual Drunkenness in America’, Journal of Studies on Alcohol, 15 (1978), 493–506, on 494.10.15288/jsa.1978.39.143344994

37

Betsy Thom , Dealing with Drink: Alcohol and Social Policy from Treatment to Management (London: Free Association, 1999), 72.

38

J.D. Sullivan and M.M. Glatt , ‘Irish Alcoholics in an English Mental Hospital’, Journal of the Irish Medical Association, 39, 223 (November 1956), 146–9.13385553

39 ‘Alcoholism among the Irish discussed by Doctor’, *Irish Times*, 2 November 1956, 5; ‘Taking a Drink’, *Irish Times*, 10 November 1956, 8.

40
*Ibid.,* 10 November 1956, 8.

41
*Ibid.*, 8.

42
*Sixty-Seventh Annual Report of the Inspectors of Lunatics (Ireland)* [CMD 32], H.C. 1919, xxv, 305, 3, 15, 35, 39.

43
*Fifty-Eighth Report of the Inspectors of Lunatics (Ireland)* [CD 4760], H.C. 1909, xxxii, 32, 17, 59. Mania *a potu* described a form of insanity attributed to alcohol consumption. Like mania itself, it was characterised by excited or violent symptoms, and sometimes identified with delirium tremens.

44

Brendan Kelly , ‘The Mental Treatment Act 1945 in Ireland: An Historical Enquiry’, History of Psychiatry, 19, 1 (2008), 47–67, on 63.1912782810.1177/0957154X06075949

45 For example, C.B. Molony, ‘The New Mental Treatment Act and its Application to General Medical Practice’, *Journal of the Medical Association of Éire*, 18, 106 (April 1946).

46 Department of Health [Ireland], *Mental Treatment Act* (Dublin: The Stationery Office, 1945), s.3. Hereafter: *Mental Treatment Act, 1945.*

47 See Butler, *op. cit.* (note 19), 29.

48 The author has surveyed admissions records for three Irish psychiatric hospitals for the period 1890–1945.

49
*Mental Treatment Act, 1945*, s.3.

50
*Ibid.*, s.184.

51
*Ibid.*, s.184.

52
*Ibid.*, s.186.

53
*Ibid*, s.189.

54 Berridge, *op. cit.* (note 2), 165; Ferriter, *op. cit.* (note 2), 144.

55 Butler, *op. cit.* (note 19), 29.

56 Shane Butler, *Alcohol, Drugs and Health Promotion in Modern Ireland* (Dublin: Institute of Public Administration, 2002), 23–4; Butler, *op. cit.* (note 19), 29–30. ‘Mental Treatment Bill, 1944’, *Journal of the Medical Association of Éire*, 16 (January–June 1945), 7–8.

57 Butler, *op. cit.* (note 56), 24–5; Butler, *op. cit.* (note 19), 30.

58 ‘Review of *Alcoholism in General Practice* by Carver, Hunt and Willcox’, *Irish Journal of Medical Science*, (1937), 89.

59
*Mental Treatment Act, 1945*, s.23.

60 Ferriter, *op. cit.* (note 8), 166, 168.

61 Ferriter, *op. cit.* (note 9), 14.

62 National Archives of Ireland, Department of Justice files, S16524 (5 June 1959); Ferriter, *op. cit.* (note 8), 190–1.

63
*Report of the Inspector of Mental Hospitals for the Year 1958* (Dublin: Stationery Office, 1960), 15.

64 Michael Viney, ‘Alcoholism in Ireland I: The Irish and the Bottle’, *Irish Times*, 6 April 1964, 8.

65
*Ibid.*, 8.

66 ‘Alcoholism not such a Grave Problem’, *Irish Press*, 18 January 1962, 4.

67 ‘Committee on Finance – Vote 54 – Health (Resumed)’, Dáil Éireann Debate, Wednesday, 9 May 1962.

68
*Ibid.*

69 ‘Action on Alcoholism’, *Irish Times*, 26 November 1964, 10.

70
*Ibid.*

71 ‘Thirst for Truth’, *Irish Times*, 27 May 1965, 9.

72 ‘National Council on Alcoholism Formed’, *Irish Times*, 28 May 1965, 1.

73 ‘Alcoholism a Major Social Problem: International Symposium opens in Dublin’, *Irish Times*, 26 May 1965, 11. The Symposium was organised by the Hospital authorities in conjunction with the International Council on Alcohol and Alcoholism, Lausanne, Switzerland. Specialists from France, Switzerland, the Netherlands, Britain and Ireland addressed this symposium, which was ‘designed mainly for doctors but is also being attended by social workers, clergy and other interested persons’.

74 ‘A Major Social Problem: European Experts Discuss Alcoholism’, *Irish Independent*, 26 May 1965, 6; ‘Minister Points to Alcoholism as Social and Medical Problem in Many Countries’, *Irish Press*, 26 May 1965, 6.

75 ‘Alcohol Research Needed in Ireland: 18,000 Victims Estimated’, *Irish Times*, 27 May 1965, 11.

76
*Report of the Commission of Inquiry on Mental Illness* (Dublin: Stationary Office, 1966), 79.

77
*Ibid.*, 80.

78
*Ibid.*, 83.

79 ‘Chief Justice Urges Action on Alcohol’, *Irish Independent*, 28 February 1967, 11.

80 Butler, *op. cit.* (note 56), 34. For more on the role of Alcoholics Anonymous in Ireland, see Butler, *op. cit.* (note 19)*.*

81

Brendan Kelly , Hearing Voices: The History of Psychiatry in Ireland (Newbridge: Irish Academic Press, 2016), 291.

82

John G. Cooney , ‘Rehabilitation of the Alcoholic’, Journal of the Irish Medical Association, 63, 396 (1970), 219–22, on 2205425070; ‘To undertake £30,000 study of Alcoholism’, *Irish Independent*, 8 February 1968, 8.

83 ‘True Nature of Alcoholism Emerging’, *Irish Examiner*, 25 November 1968, 21.

84 ‘Opening of Centre for Drug Addicts: Childers warns on abuse’, *Irish Times*, 23 December 1969, 12.

85 ‘Letter from Department of Health to Chairman of INCA dated 22 November 1971’, in Irish National Council on Alcoholism, *Alcoholism – Report to the Minister for Health* (Dublin: Three Candles Ltd, 1973), Appendix A.

86 Walsh, *op. cit.* (note 1), 65.

87
*Ibid.*

88

Dermot Walsh , ‘Alcoholism in Dublin’, Journal of the Irish Medical Association, 61, 371 (1968), 153–6.5658597

89 Dermot Walsh, ‘Alcoholism in the Republic of Ireland’, *British Journal of Psychiatry*, 115 (1969), 1021–5; Medical Correspondent, ‘High Alcoholism Rate in Ireland – Survey’, *Irish Times*, 15 October 1969, 9.

90 Lynn went on to become a Professor of Psychology at the University of Ulster from 1972 to 1995.

91

R. Lynn and Susan Hampson , ‘Alcoholism and Alcohol Consumption in Ireland’, Journal of the Irish Medical Association, 63, 392 (1970), 39–42.5413332

92

Dermot Walsh , ‘Letters to the Editor – Alcohol Consumption in Ireland’, Journal of the Irish Medical Association, 63, 395 (1970), 205.

93
*Report of the Commission of Inquiry on Mental Illness*, 80.

94 Ferriter, *op. cit.* (note 8), 191, 195.

95 Walsh, *op. cit.* (note 92), 205.

96
*Ibid.*

97
*Ibid.*

98

C.W.M. Wilson , ‘The Social Implications of Drug Use’, Journal of the Irish Medical Association, 62, 379 (1969), 4–7.5764357

99 Butler, *op. cit.* (note 56), 38.

100

John G. Cooney , ‘Alcoholism and Addiction in General Practice’, Journal of the Irish Medical Association, 53, 314 (1963), 54–7, on 55–6.14022820

101
*Ibid.*, 56.

102 Butler, *op. cit.* (note 56), 53.

103

Elizabeth Malcolm , Swift’s Hospital: A History of St Patrick’s Hospital, Dublin, 1746–1989 (Dublin: Gill and Macmillan, 1989), 288 ; For example John G. Cooney, ‘Alcoholism and Addiction in General Practice’, 54–5; John G. Cooney, ‘Alcohol and the Irish’, *Journal of the Irish Colleges of Physicians and Surgeons*, 1, 2 (1971), 54; John G. Cooney, ‘The Problem Drinker’, in K. O’Sullivan (ed.) *All in the Mind: Approaches to Mental Health* (Dublin: Gill and Macmillan, 1986); John G. Cooney, *Under the Weather: Alcohol Abuse and Alcoholism – How to Cope* (Dublin: Gill and Macmillan, 1991).

104 See, for example, Thom, *op. cit.* (note 37), 41, 74; Nicholls, *op. cit.* (note 2), 203–4; Berridge, *op. cit.* (note 2), 196.

105 Cooney, *op. cit.* (note 82), 220.

106 ‘Problem of Treating Alcoholism’, *Irish Times*, 3 March 1962, 7.

107 David Nowlan, ‘Hidden Disease Dangers: Doctors Discuss Alcohol’, *Irish Times*, 17 May 1969, 4.

108 S. J. Hanly, ‘Letter to the Editor: Stereotypes’, *Irish Times*, 1 September 1972, 16.

109 Cooney, *op. cit.* (note 102), 53.

110

Mary E. Daly , Sixties Ireland: Reshaping the Economy, State and Society, 1957–1973 (Cambridge: Cambridge University Press, 2016), 3–4, 128.

111 Ferriter, *op. cit.* (note 9), 14.

112 Cooney, *op. cit.* (note 102), 54.

113 Butler, *op. cit.* (note 56), chapter 3; Thom, *op. cit.* (note 37), 7–8.

114 Cooney, *op. cit.* (note 102), 54.

115

Diarmaid Ferriter , The Transformation of Ireland 1900–2000 (London: Profile Books, 2005), 539, 598.

116 Daly, *op. cit.* (note 110), 198–209.

117 Paul Gallagher, ‘John G. Cooney – An Appreciation’, *Irish Times*, 12 March 2018.

118 Mary McCutchan, ‘Alcoholism in Ireland’, *Irish Press*, 11 November 1968, 6.

119
*Ibid.*, 8.

120 Daly, *op. cit.* (note 110), 129.

121 Butler, *op. cit.* (note 56), 51–2.

122 Irish National Council on Alcoholism, *Alcoholism – Report to the Minister for Health* (Dublin: Three Candles Ltd, 1973), 10.

123
*Ibid.*, 45.

124 Geoffrey Dean, ‘A Need for a Philosophy in our Approach to Alcohol’, in Irish National Council on Alcoholism, *Alcoholism – Report to the Minister for Health,* Appendix M.

125
*Ibid.*, 87–90.

126

Joyce Fitzpatrick , ‘Drinking and Young People’, Alcohol and Alcoholism, 6, 3 (1971), 90–4, on 90; Joyce O’Connor, ‘Cultural Influences and Drinking Behaviour: Drinking in Ireland and England: A Tri-Ethnic Study of Drinking Among Young People and their Parents’, *Alcohol and Alcoholism*, 10, 3 (1975), 94–121.

127

Dermot Walsh , ‘Alcoholism and the Irish’, Alcohol and Alcoholism, 7, 2 (1972), 40–7, on 46; David Nowlan, ‘“Cheers” and “Slaintes” Compared’, *Irish Times*, 13 November 1975, 1.

128

Joyce O’Connor , The Young Drinkers: A Cross National Study of Social Cultural Influence (London: Tavistock, 1978).

129 O’Connor (1975), *op. cit.* (note 126), 97.

130
*Ibid.*, 98.

131 ‘Drinking Habits of Young People’, *Irish Times*, 25 November 1971, 7; Nowlan, *op. cit.* (note 127), 1; Brian McLaughlin, ‘Irish Drink Less than English, Says New Survey’, *Irish Press*, 20 May 1978, 3.

132 O’Connor (1975), *op. cit.* (note 126), 106.

133
*Ibid.*, 115.

134 Ferriter, *op. cit.* (note 8)*.*

135 Con Power, ‘Alcoholism and Irish Industry – A New Move?’, *Irish Times*, 12 September 1972, 15.

136 Walsh, *op. cit.* (note 127), 47.

137 For an illuminating discussion of similar campaigns in England, see Alex Mold , ‘From the Alcoholic to the Sensible Drinker: Alcohol Health Education Campaigns in England’, in Mark Jackson and Martin D. Moore (eds), Balancing the Self: Medicine, Politics and the Regulation of Health in the Twentieth Century (Manchester: Manchester University Press, 2020), 64–94.32286756

138 John McCarthy, ‘Government to Spend £50,000 on Anti-Drink Advt. Campaign’, *Irish Times*, 16 October 1973, 16.

139 Medical Correspondent, ‘Research Unit will Aid Campaign against Alcoholism: £150,000 Grant to St Patrick’s’, *Irish Times*, 29 March 1974, 11.

